# Comment on “K-Alpha Calculator—Krippendorff’s Alpha Calculator: A user-friendly tool for computing Krippendorff’s Alpha inter-rater reliability coefficient”: Reconciling the ratio-scale computational implementation in K-Alpha Calculator v2.0

**DOI:** 10.1016/j.mex.2026.104204

**Published:** 2026-10-13

**Authors:** Muhaimin Abdullah, Ahmad Munir, Wiwiet Eva Savitri, Widya Rizky Pratiwi, Nasrullah A

**Affiliations:** a Universitas Negeri Surabaya, Surabaya, Indonesia; b Universitas Terbuka, South Tangerang, Indonesia; c Universitas Muhammadiyah Pare-Pare, Parepare, Indonesia

Marzi, Balzano, and Marchiori introduced the K-Alpha Calculator [1], a client-side web application for computing Krippendorff's α across nominal, ordinal, interval, and ratio measurement scales, and made its full source code openly available for inspection and reuse [2]. While the browser-based tool's dependency-free design has facilitated its use across research areas, a version-specific calculation discrepancy affects the ratio-scale implementation of K-Alpha Calculator v2.0 when the minimum value of the rating scale differs from 1. The discrepancy was initially detected during the development of InStats, the corresponding author's Python-based reliability application [3]. It was subsequently verified against the Hayes and Krippendorff KALPHA SPSS reference implementation [4,5] and through inspection of the K-Alpha Calculator v2.0 source code. The K-Alpha Calculator developers were not contacted directly prior to submission of this Letter; consistent with the Letter-and-rebuttal mechanism MethodsX provides for method-related commentary, this Letter itself serves as the formal channel through which the discrepancy is first communicated to the original authors. This Letter examines the mathematical basis of the discrepancy and specifies the condition under which it occurs.

## Theoretical and mathematical basis

Krippendorff's α quantifies inter-rater reliability as the extent to which observed disagreement among raters falls below the disagreement expected under independent rating [6]. A single general expression accommodates nominal, ordinal, interval, and ratio data by substituting a level-appropriate difference function, δ²(c,k), for the distance between two ratings, a design choice grounded in the classical levels-of-measurement framework [7]: each level is defined by the class of transformations under which its numbers retain their empirical meaning, and ratio-scale measurement is distinguished from interval-scale measurement precisely by the requirement of a fixed, non-arbitrary zero point. A ratio-scale difference function is therefore expected, on theoretical grounds alone, to depend on the absolute magnitude of the values compared, not merely on their relative order or spacing — an expectation realized in Eq. (6) of the original article [1]:
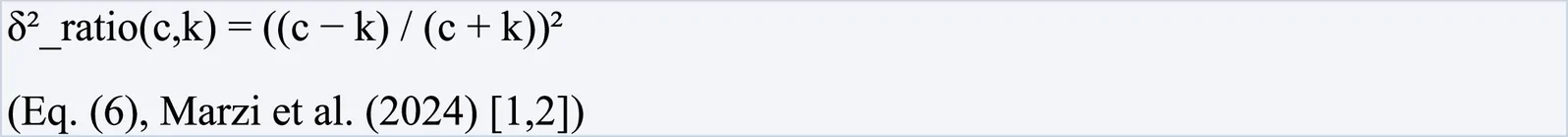
where *c* and *k* denote the observed values assigned by two raters. Because the numerator depends only on a difference, (c−k), it is unaffected by an additive shift applied to every value in a dataset: ((c+m)−(k+m)) = (c−k) for any constant *m*. The denominator, (c+k), depends on a sum and is not: ((c+m)+(k+m)) = (c+k)+2 m. A computational procedure that faithfully implements Eq. (6) is therefore expected to return a different coefficient when the entire value domain of a dataset is shifted by a nonzero constant, while an implementation found to be invariant under such a shift cannot, except by coincidence, be computing Eq. (6) as defined. This shift-sensitivity property provides an implementation-independent criterion against which the translation of Eq. (6) into source code can be assessed.

## Implementation into source code

Two independent implementations of Eq. (6) into executable code were examined. The point-estimate logic of the K-Alpha Calculator v2.0 is contained in _computeAlpha(), in src/components/Result.jsx of the tool's public repository [8]. Rather than operating on observed values directly, the implementation constructs a value-by-unit count matrix in which column position colIndex (zero-based) corresponds to the rate value minRate + colIndex, and the ratio branch computes the corresponding squared-distance term as
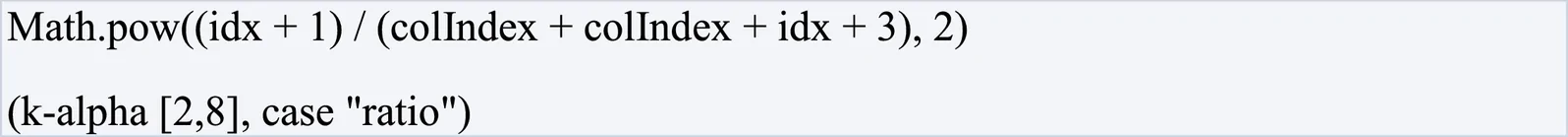


The reference implementation of Krippendorff's α published by Hayes and Krippendorff [5], distributed as the SPSS macro KALPHA, computes the same quantity in SPSS matrix language as
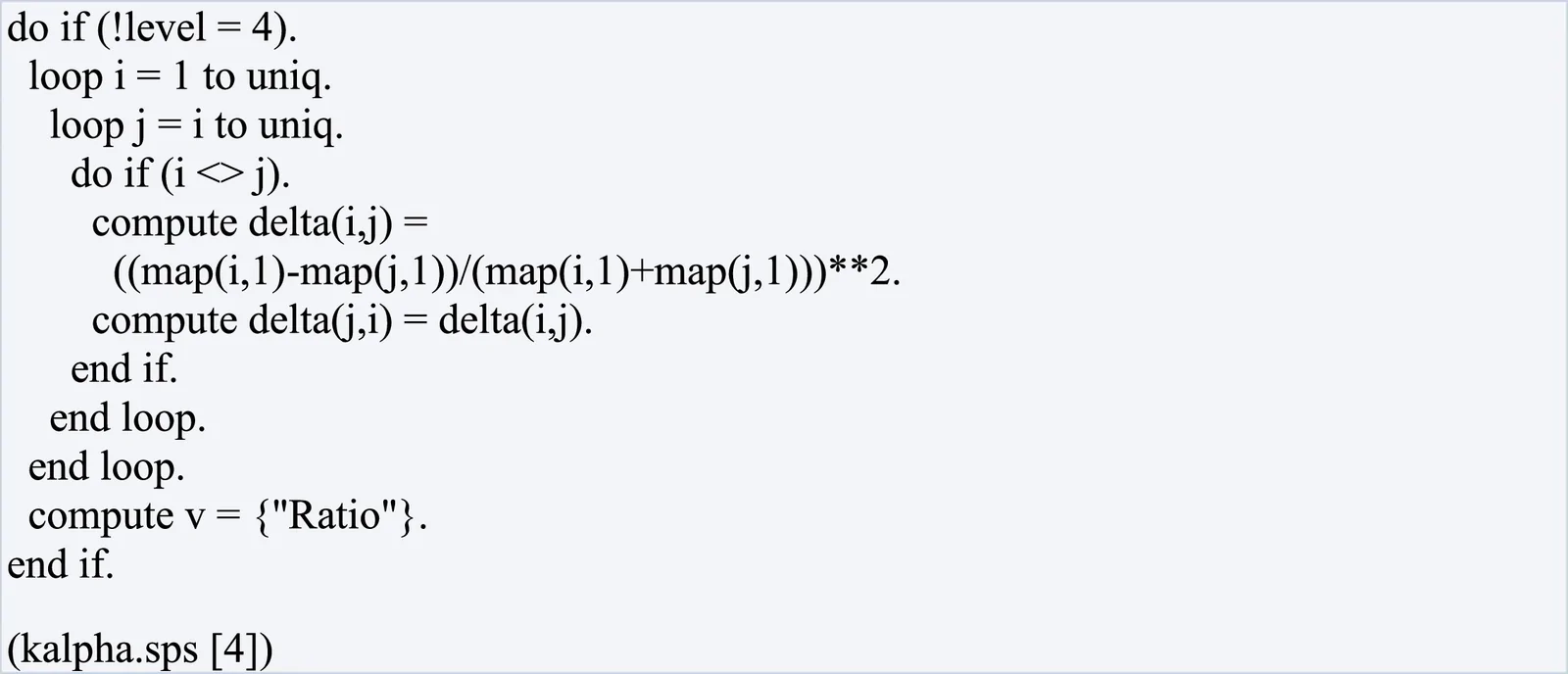
where map(i,1) and map(j,1) are the actual unique observed values in the dataset, populated earlier in the macro from the raw data rather than from loop position. This expression was used as the reference implementation for independent verification of the ratio-scale calculation in K-Alpha Calculator v2.0.

Two datasets were used for the KALPHA macro verification. For Dataset 1, the KALPHA macro was run in SPSS using:
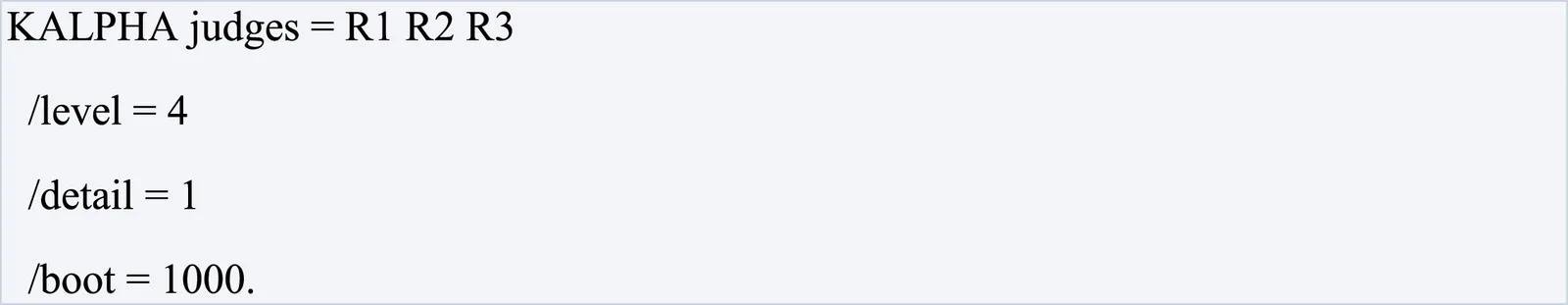


For Dataset 2, the macro was run using:
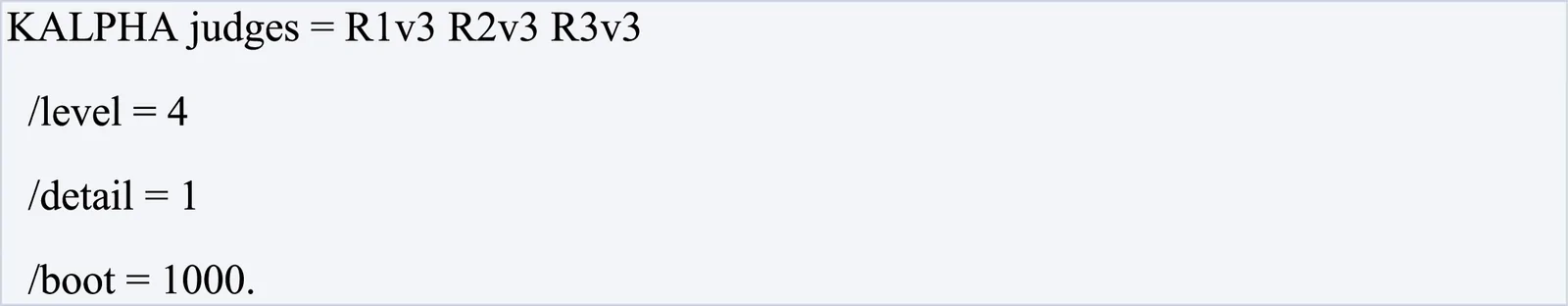


For each dataset, the KALPHA macro was downloaded from its published source [4] and run separately in SPSS using the corresponding syntax shown above. The difference in variable names reflects the structure of the two datasets, where R1, R2, and R3 are the corresponding rater variables in Dataset 1, whereas R1v3, R2v3, and R3v3 are the corresponding rater variables in Dataset 2. In both analyses, /level = 4 specifies the ratio measurement level, /detail = 1 requests detailed output, and /boot = 1000 specifies 1000 bootstrap samples. For the K-Alpha Calculator v2.0 analyses, each dataset was entered separately using the same rater assignments as those used for the corresponding KALPHA analysis, with the measurement level set to ratio.

Writing i = colIndex and j = colIndex + 1 + idx for the two positions compared in the K-Alpha Calculator v2.0 expression above, and substituting c = i+minRate, k = j+minRate, the numerator reduces to (j−i) = k−c, matching Eq. (6). The denominator, however, reduces to i+j + 2 = c+k − 2(minRate−1), which equals the denominator that Eq. (6) requires, (c+k), only when minRate = 1. The SPSS expression above contains no such condition: it computes the distance directly from map(i,1) and map(j,1), which are the observed values themselves regardless of where the value domain begins. Under the shift-sensitivity criterion, the K-Alpha Calculator's v2.0 ratio branch and the SPSS expression are therefore expected to diverge for any dataset whose minimum value of the rating scale differs from 1, and to agree, to within rounding, when it does not. Two datasets, both with a minimum value of the rating scale different from 1, were examined to confirm that the predicted discrepancy is observed in practice; they are offered as empirical confirmation of the analysis above, not as its basis.

## Empirical verification

**Dataset 1** comprised ratings from *N* = 86 units by *k* = 3 raters (values 58–96, 258 pairable ratings). In the first run, the K-Alpha Calculator v2.0 returned α = 0.379, 95% CI [0.231, 0.483]. A repeated analysis of the same dataset using the K-Alpha Calculator v2.0 yielded the same point estimate (α = 0.379) with a different 95% confidence interval of [0.270, 0.484], indicating that the reported confidence interval of v2.0 varies across repeated runs while the point estimate remains unchanged. This variability reflects the bootstrap resampling procedure itself: the K-Alpha Calculator v2.0 draws its bootstrap samples from an unseeded random number generator, so interval bounds differ slightly between runs on identical data. This behaviour is expected of any bootstrap implementation that does not fix a random seed, including the KALPHA macro, and is unrelated to the ratio-scale discrepancy examined in this Letter. The KALPHA macro, applied to the identical data with 1000 bootstrap samples, returned α = .5167, 95% CI [.4034, .6179]. The difference in point estimates reflects the ratio-scale calculation discrepancy.

**Dataset 2** comprised ratings from *N* = 52 units by *k* = 3 raters (values 59–94, 156 pairable ratings). The K-Alpha Calculator v2.0 returned α = 0.655, 95% CI [0.507, 0.759], whereas the KALPHA macro returned α = .8023, 95% CI [.7058, .8840]. Applying the interpretive thresholds reported in the original article—α ≥ 0.800 as reliable, 0.667 ≤ α < 0.800 as tentatively acceptable, and α < 0.667 as unreliable [1, p. 3; 6, p. 356]—places the two point estimates in different reliability categories: 0.655 falls below the lower threshold, whereas 0.8023 falls within the range classified as reliable. However, the 95% confidence interval for the KALPHA estimate [0.7058, 0.8840] spans the 0.800 threshold; therefore, the difference in point-estimate classification should be interpreted with caution.

In both datasets examined, the v2.0 ratio-scale expression produced a lower α estimate than the KALPHA reference implementation; however, this does not establish that the discrepancy will always be in the same direction across datasets. Similarly, the relative deviations reported in Table 1, Table 2 (26.6% and 18.4%) characterize the magnitude observed in these two datasets rather than a general property of the discrepancy; because this magnitude depends on the specific minimum value and range of each dataset, it is not expected to generalize beyond the cases examined here.

**Table 1:** K-Alpha Calculator v2.0 output compared with the KALPHA SPSS macro implementation of Eq. (6) on Dataset 1, applied at 1000 bootstrap resamples.

Dataset	K-Alpha Calculator v2.0 α [95% CI]	KALPHA (SPSS) α [95% CI]	Relative deviation
Dataset 1 (N = 86, k = 3)	0.379 [0.231, 0.483]	.5167 [.4034, .6179]	26.6%

**Note.** The K-Alpha Calculator v2.0 confidence interval represents the first run. Relative deviation was calculated as the absolute difference between the K-Alpha Calculator and KALPHA estimates divided by the KALPHA estimate and multiplied by 100%, using the KALPHA estimate as the reference value. The same calculation was used for Table 2.

**Table 2:** K-Alpha Calculator v2.0 output compared with the KALPHA SPSS macro implementation of Eq. (6) on Dataset 2, applied at 1000 bootstrap resamples.

Dataset	K-Alpha Calculator v2.0 α [95% CI]	KALPHA (SPSS) α [95% CI]	Relative deviation
Dataset 2 (N = 52, k = 3)	0.655 [0.507, 0.759]	.8023 [.7058, .8840]	18.4%

## Consequences for interpretation

The ratio-scale discrepancy can affect the interpretation of the reported coefficient when threshold-based classifications are applied. Applying the classification thresholds reported in the original article—α ≥ 0.800 as reliable, 0.667 ≤ α < 0.800 as tentatively acceptable, and α < 0.667 as unreliable [1, p. 3; 6, p. 356] — to Table 1 shows that both implementations yield values below the lower threshold for Dataset 1. Thus, although the numerical values differ, the discrepancy does not alter the resulting reliability classification. For Dataset 2 (see Table 2), the two point estimates fall into different reliability categories: 0.655 is below the lower threshold, whereas 0.8023 falls within the range classified as reliable. However, the 95% confidence interval for the KALPHA estimate [0.7058, 0.8840] spans the 0.800 threshold. Thus, the discrepancy can result in a different threshold-based classification of the reliability coefficient, although this difference in point-estimate classification should be interpreted with caution.

## Technical correction

One direct way of aligning the ratio branch with Eq. (6), consistent with the derivation above, is to restore the dependence on minRate that the denominator requires:
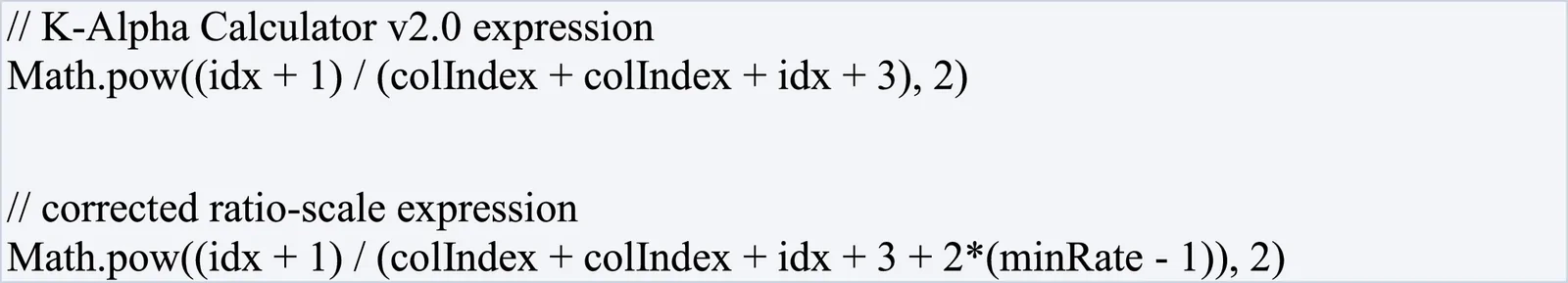


Substituting the same definitions used above, the denominator of the corrected expression reduces to i+j + 2·minRate = (c−minRate)+(k−minRate)+2·minRate = c+k for every value of minRate, matching Eq. (6) exactly rather than under a special condition; evaluated on Dataset 1, this adjustment returns α = 0.5167, matching the SPSS reference value to four decimal places.

The public availability of the K-Alpha Calculator source code enabled this independent verification of the v2.0 ratio-scale implementation. Following submission of this Letter, the K-Alpha Calculator developers independently reproduced and confirmed the discrepancy and released K-Alpha Calculator v2.1 in August 2026, which incorporates the corrected ratio-scale expression, updates the documentation, and acknowledges this Letter [8]. The developers subsequently published a formal Reply to this Letter [9], which provides their own account of the discrepancy and the v2.1 release. As of this writing, version 2.0 remains affected for ratio-scale datasets whose minimum rating value differs from 1, whereas version 2.1 incorporates the correction; the revised documentation advises researchers to recalculate prior ratio-scale analyses conducted in version 2.0 under this condition, noting that non-ratio metrics were not affected.

## Ethics statements

This Letter does not report a study involving human participants or animal subjects.

## CRediT authorship contribution statement

**Muhaimin Abdullah:** Conceptualization, Methodology, Software, Formal analysis, Investigation, Validation, Writing – original draft, Writing – review & editing. **Ahmad Munir:** Methodology, Formal analysis, Validation, Writing – review & editing, Supervision. **Wiwiet Eva Savitri:** Methodology, Formal analysis, Validation, Writing – review & editing, Supervision. **Widya Rizky Pratiwi:** Methodology, Formal analysis, Validation, Writing – review & editing, Supervision. **Nasrullah A:** Methodology, Formal analysis, Validation, Writing – review & editing, Supervision.

## Declaration of competing interest

The authors declare that they have no known competing financial interests or personal relationships that could have appeared to influence the work reported in this paper. The corresponding author develops *InStats: Reliability & Inter-rater Agreement* [3], an independently maintained, non-commercial statistical application, the initial development of which served solely as the catalyst for identifying the ratio-scale computation discrepancy in the K-Alpha Calculator. The principal claims in this Letter rest on the shift-sensitivity criterion derived from Eq. (6) and on the reference SPSS macro published by the original method's own co-author [4,5], rather than on the corresponding author’s own software alone.

## Data Availability

The datasets are available at https://doi.org/10.6084/m9.figshare.33456616.
